# HLA Class II Allele Groups Involved in Autoimmune Thyroid Diseases: Hashimoto’s Thyroiditis and Basedow–Graves Disease

**DOI:** 10.3390/life14040441

**Published:** 2024-03-27

**Authors:** Alin-Dan Chiorean, Gheorghe Zsolt Nicula, Ștefana Bâlici, Mihaela Laura Vică, Luminita-Ioana Iancu Loga, Lucia Dican, Horea Vladi Matei

**Affiliations:** 1 Department of Cell and Molecular Biology, Faculty of Medicine, “Iuliu Hațieganu” University of Medicine and Pharmacy, 400349 Cluj-Napoca, Romania; chiorean.alin@umfcluj.ro (A.-D.C.); gnicula@umfcluj.ro (G.Z.N.); sbalici@umfcluj.ro (Ș.B.); hmatei@umfcluj.ro (H.V.M.); 2 Emergency Clinical Hospital for Children, 400370 Cluj-Napoca, Romania; 3 Legal Medicine Institute Cluj-Napoca, 400006 Cluj-Napoca, Romania; 4 Clinical Institute of Urology and Renal Transplantation, 400000 Cluj-Napoca, Romania; luminisloga@yahoo.com (L.-I.I.L.); lucia.dican@umfcluj.ro (L.D.); 5 Department of Medical Biochemistry, Faculty of Medicine, “Iuliu-Hațieganu” University of Medicine and Pharmacy, 400012 Cluj-Napoca, Romania

**Keywords:** HLA, *HLA-DRB1* typing, *HLA-DQB1* typing, autoimmune thyroid diseases, Hashimoto’s thyroiditis, Basedow–Graves disease

## Abstract

Autoimmune thyroid diseases (AITD), particularly Hashimoto’s thyroiditis (HT) and Basedow–Graves disease (BGD) are diseases of global public health concern, characterized by autoimmune attacks on the thyroid gland, leading to hypothyroidism in HT and hyperthyroidism in BGD. We conducted a study between 2019 and 2021 in northwestern Transylvania (Romania) on patients with HT and with BGD compared to the control group. The aim of the study was to investigate the correlations of HLA class II alleles with AITD by identifying potential genetic susceptibility factors such as *HLA-DRB1* and *HLA-DQB1* genes in patients diagnosed with HT and BGD. Various molecular biology methods, including SSP-PCR low-resolution and PCR-SSO were employed to analyze DNA samples from patients and control subjects. Our study revealed the influence of the HLA-DRB1*03/*16 genotype as a genetic susceptibility factor for HT, a similar influence regarding BGD being observed for the HLA-DRB1*03 allele group, DRB1*03/*16 genotype, and the DRB1*03/DQB1*06 haplotype. The only protective factor detected in our study was the HLA-DRB1*13 allele group, for both HT and BGD. By elucidating any specific allele or genotype associations that might contribute to the development of AITD, our study can contribute to the prevention and early detection of these diseases.

## 1. Introduction

The most common endocrine disorders worldwide [1], thyroid dysfunctions, are diagnosed based on clinical and paraclinical investigations [2]. The key criteria for classifying pathological thyroid disorders are thyroid hormone levels and etiopathogeneses [3,4,5]. Due to various etiopathogeneses, several conditions can be distinguished: autoimmune thyroid diseases (chronic autoimmune thyroiditis and its variants [6,7], autoimmune atrophic thyroiditis, and Basedow–Graves disease [7]); iatrogenically induced thyroid diseases [8]; endemic goiter; infectious thyroiditis; and thyroid tumors [9].

Discovered in 1912 by Hakaru Hashimoto [10] and included in the family of autoimmune thyroid diseases (AITD) in 1957 [11], Hashimoto thyroiditis (HT) is the most common cause of hypothyroidism. HT is characterized by hypothyroidism in various degrees, the presence of lymphocytic infiltrates, follicular lesions, or fibrosis at glandular level, as well as in the serum detection of antibodies against thyroid peroxidase (TPO) and thyroglobulin (Tg) [12].

Basedow–Graves disease (BGD), independently described by Robert James Graves in 1835 and by Karl Adolph von Basedow in 1840 [13,14], is the most common cause of hyperthyroidism, shown to have an autoimmune cause. BGD is characterized by various symptoms (goiter, exophthalmos, tachycardia, weight loss, decreased heat tolerance, etc.) caused by an increased production of thyroxine (FT4) and triiodothyronine (T3), as a result of TSH receptors’ (TSH-R) stimulation on the surface of thyroid cells by autoantibodies mimicking the effects of the TSH hormone, resulting in the decrease of the TSH serum concentration and thyroid inflammation [15].

HT and BGD are multifactorial diseases, both environmental and genetic factors posing significant etiopathogenetic implications [16,17]. These findings were also confirmed by the studies of Brix and colleagues on a Danish sample of homozygous and dizygotic twins with autoimmune thyroid pathology, suggesting that in families with HT and BGD the transmission of each pathology is caused by the same mutations in the responsible genes [17,18,19]. The triggering of HT and BGD was significantly associated with the presence of single-nucleotide polymorphisms (SNPs) of certain genes: *PTPN22* [20], *CTLA-4* [6,21], *ZFAT* [22], *CD40* [23], *FCRL3* [24], *IL-2RA* [25], *TSHR* [26], and *TG* [27,28]. It is possible to switch from hyper- to hypothyroidism following treatment for BGD [29].

Along with SNPs, certain polymorphisms in the Human Leukocyte Antigen (HLA) genes have shown significant associations with the onset and progression of several autoimmune diseases, including HT and BGD [30,31]. The Major Histocompatibility Complex (MHC) consists of a complex of genes located on the short arm of chromosome 6 (locus 6p21) [32,33,34]. The HLA genes located in different regions of the MHC are classified into three major groups: classes I, II, and III [33]. The class I genes, which encode glycoproteins expressed on nucleated cells or on platelet membranes, playing a significant role in the presentation of antigens to Tc lymphocytes, include the classical *HLA-A*, *-B*, and *-C* genes (present in most cells) along the non-classical functional *-E*, *-F*, and *-G* genes and the non-functional pseudogenes *-H*, *-J*, and *-X* [31,33]. The class II genes encode glycoproteins expressed mainly on the membranes of antigen-presenting cells and present antigens to T-helper lymphocytes [33]. They include three groups of genes and pseudogenes controlling the processing of endogenous antigens: HLA-DR, DQ, and DP [33,35]. Class III includes genes for tumor necrosis factors (such as *TNFα*), complement components (e.g., *C3*), cytokines, and interleukins [33]. Certain proteins secreted by these genes are involved in several processes of the immune system [33,36,37]. As a result, MHC is a highly polymorphic, multigenic, multiallelic complex with loci presenting linkage disequilibrium and codominant expression [34,37]. Since the HLA region has been shown to be responsible for the modulation of the immune response, it was seen as a major candidate region in the study of genetic association with autoimmune diseases such as lupus erythematosus, psoriasis, multiple sclerosis, and for autoimmune thyroiditis in particular [31,38].

Class II genes present higher polymorphism compared to class I genes, as they are found in both α and β chains [39,40,41]. The genetic mechanisms that generate HLA sequence polymorphisms are the occurrence of point mutations or certain recombination, gene conversions, homologous and unequal crossing-over, and others [41,42]. The highest degree of polymorphism concerning the HLA molecules is observed in the variable domains involving antigen binding [42], changing the bounded peptides that influence interactions with T cell receptors [43]. The different frequency of HLA alleles in various populations helps in both investigating the function of HLA genes and the genetic differentiation between individuals [42,43,44]. Some studies conducted in the population of Transylvania, Romania, revealed the frequency of HLA profiles and their association with some pathological conditions [45,46,47].

Table 1 summarizes the results of numerous studies carried out in different populations documenting associations of *HLA-DR*, *-DQ* alleles with autoimmune thyroid diseases [38,48,49,50,51,52,53,54,55,56,57,58,59,60,61,62,63,64,65,66,67,68,69,70,71,72,73,74,75,76,77]. A 2014 study carried out on 77 subjects diagnosed with BGD between 2009 and 2011 at the Elias hospital in Bucharest, Romania, found that the HLA-DRB1*11 gene is the most frequently met HLA gene in this sample, followed by the HLA-DRB1*03 gene. So far this is the first Romanian study that demonstrated a correlation between *HLA* genes and thyroid pathology, i.e., the Basedow–Graves autoimmune disease [57].

The aim of our study was to establish correlations of *HLA-DRB1* and *-DQB1* allele groups with autoimmune thyroid diseases (HT and BGD) in the population of northwestern Romania.

## 2. Materials and Methods

### 2.1. Sample Collection

This case–control study was carried out between 2019 and 2021 in northwestern Transylvania (Romania) on 77 AITD patients (mean age 46.53 ± 11.49 years, 58 women) from the Cluj County Emergency Clinical Hospital. In total, 52 of the subjects were diagnosed with HT and 25 with BGD. The case group patients were diagnosed based on clinical criteria for hypo- or hyperthyroidism and paraclinical criteria including thyroid markers such as thyroid stimulating hormone (TSH), free thyroxine (FT4), anti-thyroid peroxidase (ATPO), anti-TSH receptor antibody and antithyroglobulin antibodies (ATG), according to the European and national guidelines for hypothyroidism and hyperthyroidism [21,22,23,24]. Thyroid goiter was observed in six cases, with seven other patients presenting thyroid atrophy. None of the HT patients reported any ophthalmological problems. The control group included 135 subjects (mean age 45.86 ± 12.95 years, 85 women) with no clinical or paraclinical manifestations of thyroid pathology or other co-morbidities tested in the Clinical Institute of Urology and Renal Transplant in Cluj-Napoca and registered in the National Registry of Voluntary Donors of Hematopoietic Stem Cells. All subjects belonging to the control group had normal reference values for TSH, FT4, and negative values of ATPO, anti-TSH receptor antibody and ATG. Matching between groups was performed according to the sex and age of the study participants.

All subjects provided informed consent forms in order to participate in the research. The present study has been approved by the ethics committee of “Iuliu Hațieganu” University of Medicine and Pharmacy in Cluj-Napoca (269/30 July 2019), based on knowledge from the principles of the Helsinki declaration of 1975 and of the 1997 European Convention of Oviedo for the protection of human rights, and the dignity of the human beings was observed.

### 2.2. Thyroid Markers Analyses

In all immunological tests, blood samples collected in sterile 6 mL anticoagulant-free vacutainers were centrifuged for 10 min at 1800× *g*. The paraclinical tests impose certain marker values as inclusion criteria for a HT diagnosis: TSH > 5.6 IU/mL, FT4 < 1.12 IU/mL, ATPO > 9 IU/mL, ATG > 10 IU/mL, and negative values for anti-TSH receptor antibodies. The inclusion criteria for diagnosis of BGD were TSH < 5.6 IU/mL, FT4 > 1.12 IU/mL, and positive values for anti-TSH receptor antibodies. All markers were determined using the chemiluminescent immunoassay method on a Unicel DXI 800 Analyzer (Beckman Coulter, Brea, CA, USA).

### 2.3. Molecular and Genetic Analyses

#### 2.3.1. DNA Extraction

From whole-blood samples collected from each subject in 2 and 3 mL sterile vacutainers with anticoagulant ethylenediaminetetraacetic acid (EDTA), DNA was extracted using a Ready DNA Spin Kit (inno-train Diagnostik GmbH, Kronberg, Germany) with silica spin filter technology. In total, 200 µL of whole blood was mixed with 200 µL of lysis buffer and 25 µL of proteinase K and incubated at 56 °C for 10 min, and then 200 µL of absolute ethanol was added. The resulting mixture was transferred to a spinning column and centrifuged for 1 min at 10,000× *g*.

To purify the DNA, consecutive DNA washes with buffer 1 (kit reagent reconstituted with absolute ethanol) and buffer 2 (kit reagent reconstituted with absolute ethanol) were performed in sterile tubes, followed by 1 min centrifugation at 10,000× *g*. After the last wash and the subsequent third centrifugation, the DNA was eluted from the spinning column and transferred at room temperature to a sterile tube in TRIS buffer (kit reagent), being subjected to a final 1 min centrifugation at 10,000× *g*. The DNA was then concentrated, its purity being analyzed using a NanoPhotometer P300 (Implen GmbH, Munich, Germany).

#### 2.3.2. HLA Typing

The *HLA-DRB1* and *HLA-DQB1* genes were typed making use of one molecular biology method for each group.

The DNA extracted from the patients belonging to the case-group was amplified using a Single Specific Primer–Polymerase Chain Reaction (SSP-PCR). The amplification of each DNA sample was performed with a low-resolution HLA-FluoGene typing kit (Inno-Train Diagnostik GmbH, Kronberg, Germany) following the manufacturer’s instructions. This set of results was evaluated with a FluoVista Analyzer (Inno-Train Diagnostik GmbH, Kronberg, Germany).

Investigation of the *HLA-DRB1* and *HLA-DQB1* genes from subjects included in the control group recruited from The National Registry of Voluntary Donors of Hematopoietic Stem Cells was performed using the Polymerase Chain Reaction Sequence–Specific Oligonucleotide (PCR-SSO) method, with a HISTO SPOT DRB1 and DQB1 kit (BAG Health Care GmbH, Lich, Germany). HLA data were analyzed with HISTO MATCH Software version 2.7.2 (BAG Health Care GmbH, Lich, Germany).

### 2.4. Statistical Analysis

The case–control groups studied by us came from northwestern Transylvania, whose population of 2,521,793 inhabitants represents 13.24% of Romania’s population of 19,053,815 inhabitants, according to the 2021 census [78]. The sample size was calculated with the Epi Info program version 7.2.0.1 (Centers for Disease Control and Prevention, Atlanta, GA, USA), taking into consideration the Fleiss method with a correction factor, and the frequencies of allele groups published by two studies. A study conducted in Romania in 2014 [57] was used for the BGD group, and a Greek study conducted in 2009 [64] was used for HT group. For the statistical analysis, Pearson’s chi-squared test and Fisher’s exact test were applied with GraphPad software, version 7.2.5.0. (Insightful Science, Atlanta, CA, USA), and associations with autoimmune thyroidian pathologies were assessed as odds ratios within 95% confidence intervals. All data were analyzed and illustrated using the Microsoft Office Excel/Word 2019 applications. Quantitative independent variables with normal distribution across groups were tested using a Student *t*-test, the null hypothesis that the variance is equal across groups being checked using Levene’s test for equality of variance.

## 3. Results

### 3.1. Clinical and Paraclinical Data

Clinical (goiter, atrophic thyroiditis, diabetes mellitus, arrhythmias, hypertension) and paraclinical (baseline free T4, baseline TSH, TPOAbs, TgAbs, Anti TSH) characteristics of our case and control groups are presented in Table 2.

### 3.2. HLA Typing Data

#### 3.2.1. HLA-DRB1 Results

The results of the cross tabulation between the dependent variable (autoimmune thyroid disease) and the independent variables (the occurrence of each of the *HLA-DRB1* allele groups) for the entire sample (77 cases and 135 controls) brought out some significant results. We found an increased frequency of the HLA-DRB1*03 allele group in the case group, while a low frequency was observed for -DRB1*13, with statistical significance as presented in Table 3.

A similar analysis regarding the association of the *HLA-DRB1* allele groups with HT was carried out for 187 individuals (52 with HT and 135 controls). No significant associations of the *-DRB1* allele groups with HT were found, but the HLA-DRB1*13 allele group was significantly more common in the control group (Table 4).

The association of *HLA-DRB1* allele groups with BGD performed for 160 individuals (25 with BGD and 135 controls) revealed a significant positive association for the HLA-DRB1*03 allele group, and a negative one for the HLA-DRB1*13 allele group, respectively (Table 5).

When analyzing the relationship between the *HLA-DRB1* genotypes and AITD, HT, and BGD, a strong association of the thyroid pathology with the *03/*16 genotype was observed (Supplementary Tables S1–S3).

#### 3.2.2. HLA-DQB1 Results

No statistically significant associations were found between *HLA-DQB1* allele groups and the studied autoimmune thyroid diseases (Table 6, Table 7 and Table 8).

HLA-DQB1 genotypes with AITD, HT, and BGD did not reveal any significant association with thyroid pathology (Supplementary Tables S4–S6).

The association of *HLA-DRB1/DQB1* haplotypes with AITD, HT, and BGD (Supplementary Tables S7–S9) revealed a significant positive association for the DRB1*03/DQB1*06 haplotype in the BGD subgroup.

The HLA susceptibility or protection factors identified in the HT and BGD subgroups are summarized in Table 9.

## 4. Discussion

Research on the correlation of HLA profiles with autoimmune thyroid pathology is in an incipient phase, and extremely different and often contradictory results are being reported in studies conducted worldwide. Such discrepancies may be attributed to differences in allelic distribution among various populations [46], the generally small samples studied to date, and to the different methods employed in the research.

Thus, a Taiwanese study on a sample of 236 BGD patients found very different HLA susceptibility genes in the Chinese population compared to local Korean and Caucasian populations: HLA-A*02:07 was shown to be the only genotype acting as a risk factor, while the HLA-A*33:03-B*58:01-DRB1*03:01 haplotype exerted a protective effect against BGD [79]. A British study on a white ethnic group of 806 patients proved the primary association of MHC class I alleles and genotypes with BGD, assigning *HLA-B* and *-C* a much more important role in the etiopathogenesis of BGD than the secondary association with MHC class II caused by linkage disequilibrium [80].

Another study conducted in the Czech Republic indicated that the HLA-DQA1*05 allele group and the HLA DRB1*03-DQA1*05-DQB1*02 haplotype presented significantly higher frequencies in a group of 206 BGD patients than in the general population, while the HLA-DQA1*05 allele group was significantly associated with relapse or unsuccessful long-term conservative treatment [81]. In the Greek population, the frequencies of HLA-DRB1*04:05, -DQB1*02:01, -DQB1*03:02, and -DQA1*03:01 were significantly higher in the group of 125 HT patients than those in the control group with 500 healthy individuals, while the frequency of HLA-DRB1*07, -DQB1*05:02 and -DQB1*06:04 were significantly lower [64].

Our study indicates that the HLA-DRB1*03 allele group acts as a susceptibility factor for BGD, as confirmed by studies from Romania [57], Poland [61], Tunisia [60], Florida, and Canada [58]. Thus, a Polish study performed on 159 patients demonstrated for the first time strong associations between HLA alleles and BGD, finding that -DRB1*01:03, -DRB1*03:01, -DRB1*11:01, -DRB1*13:03, -DRB1*14:01, -DQB1*02:01, and -DQB1*03:01 were genetic markers of increased risk, while the -DRB1*07:01, -DQB1*02:02, and -DQB1*03:03 alleles exerted a protective influence [61]. Susceptibility similarities in patients from Romania and North America also supported by our study, can be explained by the Caucasian origin of the analyzed groups, even if they belong to two different continents. A case report from Tunisia suggests that the HLA-DRB1*03 allele group may be the common cause of three simultaneous diseases, schizophrenia, Graves’ disease, and type 2 diabetes [60]. On the other hand, a study on 625 white Caucasian HT patients from the UK showed a predisposing effect for HT detected at -DRB1*03 [55], while research performed on 235 patients with AITD in south India found the protective effects of the -DRB1*03 allele group against HT [56].

Our study highlighted the protective influence of the HLA-DRB1*13 allele group for both HT and BGD. Several studies conducted in Japan, South Korea, and the UK [50,51,54,55] reached similar conclusions in regard to HT or BGD. Thus, a study investigating 82 patients with HT revealed that the HLA-DRB1*13:02 allele has a protective role against HT [50]. Another study conducted in Japan on 547 patients with BGD confirms the HLA-DRB1*13 gene (more precisely the HLA-DRB1*13:02 allele) as an epistatic factor to the *HLA-DP5* gene which is a known susceptibility factor for the disease [51]. The same study performed on 444 patients with HT found significant effects of the HLA-DRB1*13:02 allele against the development of HT [51]. Research on the association of *HLA-DR* genes with BGD in 198 patients from South Korea revealed the protective role of the HLA-DRB1*13:02 allele against the disease [54]. Analysis of HLA class II genes in 625 white Caucasian HT patients in the UK revealed the protective effects of the HLA-DRB1*13 allele group against the development of HT [55].

We were unable to demonstrate that *HLA-DQB1* allele groups/alleles act as either protective or susceptibility factors for HT and/or BGD. However, numerous studies involving varied populations have identified several *HLA-DRB1* and *-DQB1* allele groups/alleles as risk or protective factors for HT or BGD, as summarized in Table 1. Several studies indicate that the HLA-DRB1*04, *08, and *09 allele groups [48,50,55,66], and the HLA-DQB1*03, *04 and *06 allele groups [55,63,64,66], respectively, act as risk factors for HT. On the other hand, HLA-DRB1*03, *08 and *09 [38,48,54,57,58,59,60,61,66], along with the HLA-DQB1*03, *05 and *06 allele groups [49,51,54,65,68,69,71,72,73], were found to be risk factors for BGD. In contrast, the HLA-DRB1*03 and *10 allele groups [56], along with the HLA-DQB1*03, *05 and *06 allele groups [51,55,64,67,77], were identified as protective factors against HT, with a similar influence in regard to BGD being observed for the HLA-DRB1*01 and *12 [50,51,52,53,54,70,73], and HLA-DQB1*05 and *06 allele groups [51,52,53,54,73,76,77], respectively.

Another line of research hypothesized that a linkage disequilibrium between certain genetic polymorphisms and HLA genotypes or haplotypes could help identifying genetic markers for BGD or HT.

Thus, a study carried out in the UK highlighted that the association of the large multifunctional proteasome 2 (LMP 2) locus with BGD is due to a linkage disequilibrium with the associated *HLA* haplotype DRB1*03:04-DQB1*02-DQA1*05:01 [82]. An American study highlighted that the substitution of alanine or glycine with arginine at position 74 of the HLA-DRB1*03 gene increases the risk of BGD, allowing the pathogenic peptides TSHR and/or thyroglobulin to be presented to T cells [38]. Confirmation of this mechanism will open the possibility of therapeutic interventions by blocking the presentation of pathogenic peptides to T cells.

A recent review that analyzed a large number of studies conducted on all continents highlights the polygenic inheritance of BGD, with more than 80 HLA and non-HLA susceptibility loci identified to date, as well as the need for large cohort studies to certify the association of known and unknown genetic variants with relevant clinical phenotypes (age of onset, goiter size, thyrotoxicosis severity, ophthalmopathy, or post-therapeutic relapse) [83]. Our study expands the research in the Romanian population regarding the genetic factors of susceptibility and protection against autoimmune thyroid diseases.

The limits of our study are given by the small sample size, respectively, using the low resolution of the typing method. The size of each case group from our study could not reach the initially calculated sample size due to some objective restrictive conditions under which the study was conducted, such as the low prevalence of the disease [84], the COVID-19 pandemic that occurred during the study period, involving the impossibility of patients to be admitted to clinics and consequently the impossibility to collect samples from such patients, and the limited study period. The deviation from the estimated number of required cases was compensated by recruiting a larger number of controls than initially anticipated, a strategy adopted to maximize the accuracy and validity of the statistical analysis. Consequently, the low-resolution HLA typing method does not allow us to differentiate between the DRB1*03:01 and DRB1*03:02 alleles. Our discovery that the DRB1*03/DQB1*06 haplotype is a susceptibility factor for BGD, with a borderline statistical significance of 0.049, could be confirmed or refuted in future studies with larger samples. In Romania’s population, among the most common alleles and allele group are DRB1*03:01, DRB1*07:01, DRB1*15:01, and DRB1*13 [44]. It is known that DRB1*03 and DRB1*07, due to linkage disequilibrium, frequently pair with DQB1*02 [55]. Because patients inherit two alleles, the DRB1*03 and DRB1*07 allele groups often combining with the DRB1*13 or DRB1*15 allele groups, which, in turn, is more frequently associated with DQB1*06 through linkage disequilibrium [55]. This complexity underscores how linkage disequilibrium can influence disease risk by combining allele groups/alleles that are not necessarily common, creating a genetic mosaic that affects immune system function and predisposes individuals to specific conditions.

Further studies on larger samples are needed to enhance knowledge concerning the influence of HLA alleles, genotypes, and haplotypes on autoimmune thyroid diseases, as well as the interactions between specific genes and *HLA* genes with the aim of identifying genetic markers for BGD and HT.

## 5. Conclusions

To conclude, our study revealed the influence of the HLA-DRB1*03/*16 genotype as a genetic susceptibility factor for HT, with a similar influence regarding BGD being observed for the HLA-DRB1*03 allele group, the -DRB1*03/*16 genotype, and the -DRB1*03/-DQB1*06 haplotype. The only protective factor detected in our study was the HLA-DRB1*13 allele group, for both HT and BGD.

Further studies on larger samples are needed to enhance knowledge concerning the influence of HLA class II genes on autoimmune thyroid diseases, as well as the interactions between specific genes and HLA genes with the aim of identifying genetic markers for BGD and HT.

## Figures and Tables

**Table 1:** HLA class II allele groups/alleles associated with autoimmune thyroid pathology (a literature review).

HLA ^1^ Allele Groups/Alleles	Susceptibility Factor for HT ^2^	Protection Factor against HT	Susceptibility Factor for BGD ^3^	Protection Factor against BGD
	Country/Sample/ [Reference]	Country/Sample/ [Reference]	Country/Sample/ [Reference]	Country/Sample/ [Reference]
*HLA-DRB1*				
HLA-DRB1*01		South Korea/73/[48]		
HLA-DRB1*01:01			South Africa/63/[49]	Japan/325/[50], 991/[51],
				100/[52], 76/[53]
				South Korea/198/[54]
HLA-DRB1*01:03			Poland/159/[61]	
HLA-DRB1*03	UK/625/[55]	India/235/[56]	Romania/77/[57]	
	Spain/90/[59]		Florida and Canada/92/[58]	
			USA/Review/[38] Spain/90/[59]	
			Tunisia/78/[60]	
HLA-DRB1*03:01			Poland/159/[61]	
HLA-DRB1*04	South Korea/73/[48]		Italy/58[62]	
	UK/625/[55]			
HLA-DRB1*04:03	Japan/71/[63]			
HLA-DRB1*04:05	Greece/125/[64]		Japan/325/[50], 991/[51]	
HLA-DRB1*07		UK/625/[55]		South Korea/73/[48]
		Greece/125/[64]		Thailand/124/[65]
HLA-DRB1*07:01				Poland/159/[61]
HLA-DRB1*08	UK/625/[55]		South Korea/73/[48]	
HLA-DRB1*08:02			Japan/29/[66]	
HLA-DRB1*08:03	Japan/325/[50],		Japan/29/[66], 62/[68]	
	991/[51], 82/[67]		South Korea/198/[54]	
HLA-DRB1*09:01	Japan/325/[50],		Hong Kong/97/[69]	
	29/[66], 82/[67]		Taiwan/241/[70]	
			USA/45/[71]	
HLA-DRB1*10		India/235/[56]		
HLA-DRB1*11	India/235/[56]		Romania/77/[57]	
HLA-DRB1*11:01			Poland/159/[61] USA/133/[72]	
HLA-DRB1*12	India/235/[56]			
HLA-DRB1*12:02				South Korea/198/[54]
				Taiwan/241/[70], 499/[73]
HLA-DRB1*13:02		Japan/325/[50], 991/[51], 82/[67] UK/625/[55]		Japan/991/[51] South Korea/198/[54]
HLA-DRB1*13:03			Poland/159/[61]	
HLA-DRB1*14:01			Poland/159/[61]	
HLA-DRB1*14:03			Japan/325/[50], 991/[51], 62/[68]	
HLA-DRB1*14:04	India/48/[74]			
HLA-DRB1*15:01		Japan/82/[67]	Taiwan/499/[73]	
HLA-DRB1*15:02				
				Japan/325/[50], 991/[51]
HLA-DRB1*16	Greece/17/[75]		Brazil/242/[76]	
HLA-DRB1*16:02			South Korea/198/[54]	
			Taiwan/499/[73]	
			Thailand/124/[65]	
***HLA-DQB1***				
HLA-DQB1*02:01	Greece/125/[64]		Poland/159/[61]	
HLA-DQB1*02:02				Poland/159/[61]
HLA-DQB1*03:01	UK/625/[55]	Iraq/30/[77]	Poland/159/[61] Japan/991/[51], 62/[68]	Iraq/30/[77]
			USA/133/[72]	
HLA-DQB1*03:02	Greece/125/[64]			
HLA-DQB1*03:03	Japan/71/[63], 29/[66], 82/[67]		Hong Kong/97/[69]	Poland/159/[61]
			USA/45/[71]	
HLA-DQB1*04:01	Greece/125/[64]		Japan/991/[51]	
HLA-DQB1*04:02	UK/625/[55]			
HLA-DQB1*05:01			South Africa/63/[49]	Japan/991/[51], 100/[52], 76/[53]
				South Korea/198/[54]
				Taiwan/499/[73]
HLA-DQB1*05:02		Greece/125/[64]	South Korea/198/[54]	
			Taiwan/499/[73]	
			Thailand/124/[65]	
HLA-DQB1*06:01	Japan/82/[67]	Iraq/30/[77]	South Korea/198/[54]	Iraq/30/[77]
			Japan/62/[68]	Japan/991/[51]
HLA-DQB1*06:02		Japan/82/[67]	Taiwan/499/[73]	
HLA-DQB1*06:04		Japan/991/[51], 82/[67]		Japan/991/[51]
		UK/625/[55]		South Korea/198/[54]
		Greece/125/[64]		

^1^ HLA, human leukocyte antigen; ^2^ HT, Hashimoto’s thyroiditis; ^3^ BGD, Basedow–Graves’ disease.

**Table 2:** Clinical and paraclinical characteristics of patients with HT, BGD, AITD, and control group.

Variable ^1^	HT ^2^ Patients (n ^5^ = 52)	BGD ^3^ Patients (n = 25)	AITD ^4^ (HT + BGD) Patients (n = 77)	Control Subjects (n = 135)
Female gender	40 (76.92)	18 (72)	58 (75.32)	85 (62.96)
Male gender	12 (23.08)	7 (28)	19 (24.68)	50 (37.04)
Age	48 ± 10.95	44 ± 11.89	46.53 ± 11.49	45.86 ± 12.95
Goiter	8 (15.38)	4 (16)	12 (15.58)	0
Atrophic thyroiditis	8 (15.38)	0	8 (10.39)	0
Diabetes mellitus	4 (7.69)	0	4 (5.19)	0
Arrhythmias	1 (1.92)	4 (16)	5 (6.49)	0
Hypertension	2 (3.84)	1 (4)	3 (3.89)	6 (4.44)
Baseline FT4 ^6^ (ng/dL)	3.64 ± 5.11	8.29 ± 7.81	4.57 ± 6.52	0.80 ± 0.13
Baseline TSH ^7^ (mU/mL)	8.62 ± 24.90	2.79 ± 6.23	6.75 ± 21.01	2.65 ± 1.19
TPOAbs ^8^ (mU/mL)	216.57 ± 649.07	54.88 ± 140.94	262.39 ± 563.71	3.94 ± 2.59
TgAbs ^9^ (mU/mL)	80.75 ± 815.33	6.72 ± 13.11	161.74 ± 704.60	2.25 ± 0.71
Anti TSH (positive)	0	25	25	0

^1^ Hormone concentrations, antibodies’ concentrations and age of the subjects were calculated as means ± standard deviation, while all the other results were presented as count (percentage); ^2^ HT, Hashimoto’s thyroiditis; ^3^ BGD, Basedow–Graves’ disease; ^4^ AITD, autoimmune thyroid disease; ^5^ n, persons number; ^6^ FT4, free thyroxine, reference values: <1.12 IU/mL for HT group and >1.12 IU/mL for BGD group; ^7^ TSH, free triiodothyronine, reference values: >5.6 IU/mL for HT group and <5.6 IU/mL for BGD group; ^8^ TPOAbs, thyroid peroxidase antibodies, reference values: >9 IU/mL for HT group; ^9^ TgAbs, thyroglobulin antibodies, reference values: >10 IU/mL for HT group.

**Table 3:** Logistic regressions predicting AITD group compared to control, based on *HLA-DRB1* allele groups.

*HLA* ^1^*-DRB1* Allele Groups	AITD ^2^ Group (n ^3^ = 77)/154 ^5^		Control Group (n = 135)/270 ^6^		OR ^7^ (95% CI ^8^)	*RR* ^9^ (95% CI)	*p*
	No. ^4^	%	No.	%			
*01	19	12.34	28	10.37	1.23 (0.66–2.29)	1.14 (0.78–1.65)	0.52
*03	**21**	**13.64**	**19**	**7.04**	**2.08 (1.08–4.04)**	**1.51 (1.09–2.1)**	**0.03**
*04	14	9.09	25	9.26	0.98 (0.5–1.95)	0.98 (0.63–1.53)	1
*07	13	8.44	30	11.11	0.7 (0.37–1.46)	0.81 (0.5- 1.31)	0.4
*08	3	1.95	3	1.11	1.76 (0.35–0.8)	1.38 (0.61–3.11)	0.67
*09	2	1.30	2	0.74	1.76 (0.24–12.65)	1.38 (0.51–3.7)	0.62
*10	1	0.65	4	1.48	0.43 (0.04–3.9)	0.54 (0.09–3.17)	0.65
*11	33	21.43	60	22.22	0.95 (0.59–1.54)	0.97 (0.71–1.32)	0.9
*12	0	0.00	2	0.74	0.34 (0.01–7.2)	NC ^10^	0.53
*13	**8**	**5.19**	**41**	**15.19**	**0.306 (0.14–0.67)**	**0.42 (0.22–0.8)**	**0.015**
*14	5	3.25	9	3.33	0.97 (0.32–2.95)	0.98 (0.48–2)	1
*15	15	9.74	21	7.78	1.28 (0.63–2.56)	1.16 (0.77–1.75)	0.47
*16	20	12.99	26	9.63	1.4 (0.75–2.6)	-	0.33

^1^ HLA, human leukocyte antigen; ^2^ AITD, autoimmune thyroid disease; ^3^ n, persons number; ^4^ No., allele number; ^5,6^ total number of alleles in the analyzed group; ^7^ OR, odds ratio; ^8^ CI, confidence interval; ^9^ RR, risk ratio; ^10^ NC, cannot be computed.

**Table 4:** Logistic regressions predicting HT subgroup compared to control, based on *HLA-DRB1* allele groups.

*HLA* ^1^*-DRB1* Allele Groups	HT ^2^ Group (n ^3^ = 52)/104 ^5^		Control Group (n = 135)/270 ^6^		OR ^7^ (95% CI ^8^)	*RR* ^9^ (95% CI)	*p*
	No. ^4^	%	No.	%			
*01	14	13.46	28	10.37	1.34 (0.67–2.66)	1.23 (0.77–1.95)	0.46
*03	11	10.58	19	7.04	1.56 (0.71–3.4)	1.25 (0.82–2.23)	0.29
*04	9	8.65	25	9.26	0.92 (0.41–2.06)	0.94 (0.52–1.7)	1
*07	8	7.69	30	11.11	0.66 (0.29–1.5)	0.73 (0.38–1.39)	0.44
*08	2	1.92	3	1.11	1.74 (0.28–10.6)	1.44 (0.48–4.2)	0.62
*09	2	1.92	2	0.74	2.62 (0.36–18.91)	1.81 (0.67–4.9)	0.3
*10	1	0.96	4	1.48	0.64 (0.07–5.8)	0.71 (0.12–4.17)	1
*11	24	23.08	60	22.22	1.05 (0.61–1.8)	1.03 (0.7–1.52)	0.89
*12	0	0	2	0.74	0.51 (0.02–10.8)	NC ^10^	1
*13	**6**	**5.77**	**41**	**15.19**	**0.34 (0.14–0.83)**	**0.42 (0.19–0.9)**	**0.01**
*14	3	2.89	9	3.33	0.86 (22–3.24	0.89 (0.33–2.42)	1
*15	12	11.54	21	7.78	1.54 (0.73–3.2)	1.34 (0.83–2.18)	0.3
*16	12	11.54	26	9.63	1.22 (0.59–2.52)	-	0.57

^1^ HLA, human leukocyte antigen; ^2^ HT, Hashimoto’s thyroiditis; ^3^ n, persons number; ^4^ No., allele number; ^5,6^ total number of alleles in the analyzed group; ^7^ OR, odds ratio; ^8^ CI, confidence interval; ^9^ RR, risk ratio; ^10^ NC, cannot be computed.

**Table 5:** Logistic regressions predicting BGD subgroup compared to control, based on *HLA-DRB1* allele groups.

*HLA* ^1^*-DRB1* Allele Groups	BGD ^2^ Group (n ^3^ = 25)/50 ^5^		Control Group (n = 135)/270 ^6^		OR ^7^ (95% CI ^8^)	*RR* ^9^ (95% CI)	*p*
	No. ^4^	%	No.	%			
*01	5	10.00	28	10.37	0.96 (0.35–2.6)	0.96 (0.41–2.26)	1
*03	10	20.00	19	7.04	**3.3 (1.43–7.6)**	**2.5 (1.4–4.47)**	**0.006**
*04	5	10.00	25	9.26	1.09 (0.39–2.99)	1.07 (0.46–2.49)	0.79
*07	5	10.00	30	11.11	0.88 (0.32–2.41)	0.9 (0.38–2.12)	1
*08	1	2.00	3	1.11	1.81 (0.18–17.83)	1.61 (0.29–8.9)	0.49
*09	0	0.00	2	0.74	1.06 (0.05–22.5)	NC ^10^	1
*10	0	0.00	4	1.48	0.58 (0.03–11.07)	NC	1
*11	9	18.00	60	22.22	0.76 (0.35–1.67)	0.8 (0.4–1.56)	0.57
*12	0	0.00	2	0.74	1.06 (0.05–22.5)	NC	1
*13	2	4.00	41	15.19	**0.23 (0.05–0.99)**	**0.26 (0.06–1.06)**	**0.03**
*14	2	4.00	9	3.33	1.2 (0.25–5.7)	1.17 (0.32–4.2)	0.68
*15	3	6.00	21	7.78	0.75 (0.21–2.6)	0.78 (0.26–2.38)	1
*16	8	16.00	26	9.63	1.78 (0.75–4.21)	-	0.2

^1^ HLA, human leukocyte antigen; ^2^ BGD, Basedow–Graves’ disease; ^3^ n, persons number; ^4^ No., allele number; ^5,6^ total number of alleles in the analyzed group; ^7^ OR, odds ratio; ^8^ CI, confidence interval; ^9^ RR, risk ratio; ^10^ NC, cannot be computed.

**Table 6:** Logistic regressions predicting AITD group compared to control, based on *HLA-DQB1* allele groups.

*HLA* ^1^*-DQB1* Allele Groups	AITD ^2^ Group (n ^3^ = 77)/154 ^5^		Control Group (n = 135)/270 ^6^		OR ^7^ (95% CI ^8^)	*RR* ^9^ (95% CI)	*p*
	No. ^4^	%	No.	%			
*02	31	20.13	40	14.81	1.44 (0.86–2.43)	1.25 (0.92–1.69)	0.17
*03	54	35.06	109	40.37	0.81 (0.53–1.22)	0.87 (0.67–1.14)	0.35
*04	2	1.30	2	0.74	1.76 (0.24–12.65)	1.38 (0.51–3.70)	0.62
*05	46	29.87	68	25.19	1.26 (0.81–1.96)	1.15 (0.88–1.51)	0.3
*06	21	13.64	51	18.89	0.67 (0.39–1.17)	0.77 (0.52–1.13)	0.18

^1^ HLA, human leukocyte antigen; ^2^ AITD, autoimmune thyroid disease; ^3^ n, persons number; ^4^ No., allele number; ^5,6^ total number of alleles in the analyzed group; ^7^ OR, odds ratio; ^8^ CI, confidence interval; ^9^ RR, risk ratio.

**Table 7:** Logistic regressions predicting HT subgroup compared to control, based on *HLA-DQB1* allele groups.

*HLA* ^1^*-DQB1* Allele Groups	HT ^2^ Group (n ^3^ = 52)/104 ^5^		Control Group (n = 135)/270 ^6^		OR ^7^ (95% CI ^8^)	*RR* ^9^ (95% CI)	*p*
	No. ^4^	%	No.	%			
*02	18	17.31	40	14.81	1.2 (0.45–1.49)	1.14 (0.74–1.74)	0.52
*03	38	36.54	109	40.37	0.85 (0.53–1.35)	0.88 (0.63–1.25)	0.55
*04	1	0.96	2	0.74	1.3 (0.11–14.51)	1.2 (0.24–6.00)	1
*05	31	29.81	68	25.19	1.26 (0.76–2.08)	1.18 (0.82–1.67)	0.36
*06	16	15.38	51	18.89	0.7 (0.42–1.44)	0.83 (0.52–1.32)	0.45

^1^ HLA, human leukocyte antigen; ^2^ HT, Hashimoto’s thyroiditis; ^3^ n, persons number; ^4^ No., allele number; ^5,6^ total number of alleles in the analyzed group; ^7^ OR, odds ratio; ^8^ CI, confidence interval; ^9^ RR, risk ratio.

**Table 8:** Logistic regressions predicting BGD subgroup compared to control, based on *HLA-DQB1* allele groups.

*HLA* ^1^*-DQB1* Allele Groups	BGD ^2^ Group (n ^3^ = 25)/50 ^5^		Control Group (n = 135)/270 ^6^		OR ^7^ (95% CI ^8^)	*RR* ^9^ (95% CI)	*p*
	No. ^4^	%	No.	%			
*02	13	26.00	40	14.81	2 (0.98–4.1)	1.77 (1.01–3.09)	0.06
*03	16	32.00	109	40.37	0.69 (0.36–1.32)	0.73 (0.422–1.27)	0.34
*04	1	2.00	2	0.74	2.7 (0.24–30.76)	2.1 (0.42–10.9)	10.9)
*05	15	30.00	68	25.19	1.27 (0.65–2M47)	1.22 (0.7–2.12)	0.48
*06	5	10.00	51	18.89	0.47 (0.18–1.26)	0.53 (0.21–1.26)	0.15

^1^ HLA, human leukocyte antigen; ^2^ BGD, Basedow–Graves’ disease; ^3^ n, persons number; ^4^ No., allele number; ^5,6^ total number of alleles in the analyzed group; ^7^ OR, odds ratio; ^8^ CI, confidence interval; ^9^ RR, risk ratio.

**Table 9:** Susceptibility and protection factors in the AITD group, respectively, in HT and BGD subgroups.

HLA ^1^ Markers	Susceptible for AITD ^2^	Protective for AITD	Susceptible for HT ^3^	Protective for HT	Susceptible for BGD ^4^	Protective for BGD
***HLA-DRB1* allele groups**	*03	*13	**-**	*13	*03	*13
***HLA-DQB1* allele groups**	**-**	**-**	**-**	**-**	**-**	**-**
***HLA-DRB1* genotypes**	*03/*16	**-**	*03/*16	**-**	*03/*16	**-**
***HLA-DQB1* genotypes**	**-**	**-**	**-**	**-**	**-**	**-**
***HLA-DRB1/DQB1* haplotypes**	**-**	**-**	**-**	**-**	DRB1*03/DQB1*06	**-**

^1^ HLA, human leukocyte antigen; ^2^ AITD, autoimmune thyroid disease; ^3^ HT, Hashimoto’s thyroiditis; ^4^ BGD, Basedow–Graves’ disease.

## Data Availability

The data included in this paper are available from the corresponding author. Data are not publicly available due to patient privacy.

## Supplementary Materials

The following supporting information can be downloaded at https://www.mdpi.com/article/10.3390/life14040441/s1. Comparison between autoimmune thyroid diseases (AITD), Hashimoto’s thyroiditis (HT), Basedow–Graves disease (BGD), and control groups, based on genotypes resulting from HLA-DRB1 typing: Table S1: Logistic regressions predicting AITD group compared to control, based on HLA-DRB1 genotypes; Table S2: Logistic regressions predicting HT subgroup compared to control, based on HLA-DRB1 genotypes; Table S3: Logistic regressions predicting BGD subgroup compared to control, based on HLA-DRB1 genotypes; Comparison between autoimmune thyroid diseases (AITD), Hashimoto’s thyroiditis (HT), Basedow–Graves disease (BGD) and control groups, based on genotypes resulting from HLA-DQB1 typing: Table S4: Logistic regressions predicting AITD group compared to control, based on HLA-DQB1 genotypes; Table S5: Logistic regressions predicting HT subgroup compared to control, based on HLA-DQB1 genotypes; Table S6: Logistic regressions predicting BGD subgroup compared to control, based on HLA-DQB1 genotypes; Comparison between autoimmune thyroid diseases (AITD), Hashimoto’s thyroiditis (HT), Basedow-Graves disease (BGD) and control groups, based on HLA-DRB1/DQB1 haplotypes resulting: Table S7: Logistic regressions predicting AITD group compared to control, based on HLA-DRB1/DQB1 haplotypes; Table S8: Logistic regressions predicting HT subgroup compared to control, based on HLA- DRB1/DQB1 haplotypes; Table S9: Logistic regressions predicting BGD subgroup compared to control, based on HLA- DRB1/DQB1 haplotypes.
