# Supplementary material for: HLA Class II Allele Groups Involved in Autoimmune Thyroid Diseases: Hashimoto’s Thyroiditis and Basedow–Graves Disease
Source: Life (Basel). 2024 Mar 27;14(4):441. doi: 10.3390/life14040441 (PMC11050925; doi:10.3390/life14040441)
Supplement: Supplementary file 1 [file life-14-00441-s001.zip › life-2892815-supplementary.pdf]

HLA Class II Allele Groups Involved in Autoimmune Thyroid Diseases: Hashimoto's Thyroiditis and Basedow–Graves Disease

Alin-Dan Chiorean [1,2], Gheorghe Zsolt Nicula [1], Ștefana Bâlici [1], Mihaela Laura Vică [1,3,*], Luminita-Ioana Iancu Loga [4], Lucia Dican [4,5] and Horea Vladi Matei [1,3]

[1] Department of Cell and Molecular Biology, Faculty of Medicine, "Iuliu Hațieganu" University of Medicine and Pharmacy, 400349 Cluj-Napoca, România; Chiorean.Alin@umfcluj.ro (A.D.C.); gnicula@umfcluj.ro (G.Z.N.); sbalici@umfcluj.ro (Ș.B.); mvica@umfcluj.ro (M.L.V.); hmatei@umfcluj.ro (H.V.M.)
[2] Emergency Clinical Hospital for Children, 400370 Cluj-Napoca, România
[3] Institute of Legal Medicine Cluj-Napoca, 400006 Cluj-Napoca, România
[4] Clinical Institute of Urology and Renal Transplantation, 400000 Cluj-Napoca, Romania; luminisloga@yahoo.com (L.I.I.L.)
[5] Department of Medical Biochemistry, Faculty of Medicine, "Iuliu-Hațieganu" University of Medicine and Pharmacy, 400012 Cluj-Napoca, Romania; lucia.dican@umfcluj.ro (L.D.)
* Correspondence: mvica@umfcluj.ro (M.L.V.)

Supplemental Table of Contents

Comparison between autoimmune thyroid diseases (AITD), Hashimoto's thyroiditis (HT), Basedow-Graves disease (BGD) and control groups, based on genotypes resulting from HLA-DRB1 typing 2
Table S1. Logistic regressions predicting AITD group compared to control, based on HLA-DRB1 genotypes 2
Table S2. Logistic regressions predicting HT subgroup compared to control, based on HLA-DRB1 genotypes 3
Table S3. Logistic regressions predicting BGD subgroup compared to control, based on HLA-DRB1 genotypes 4
Comparison between autoimmune thyroid diseases (AITD), Hashimoto's thyroiditis (HT), Basedow-Graves disease (BGD) and control groups, based on genotypes resulting from HLA-DQB1 typing 6
Table S4. Logistic regressions predicting AITD group compared to control, based on HLA-DQB1 genotypes 6
Table S5. Logistic regressions predicting HT subgroup compared to control, based on HLA-DQB1 genotypes 6
Table S6. Logistic regressions predicting BGD subgroup compared to control, based on HLA-DQB1 genotypes 7
Comparison between autoimmune thyroid diseases (AITD), Hashimoto's thyroiditis (HT), Basedow-Graves disease (BGD) and control groups, based on HLA-DRB1/DQB1 haplotypes resulting 8
Table S7. Logistic regressions predicting AITD group compared to control, based on HLA-DRB1/DQB1 haplotypes 8
Table S8. Logistic regressions predicting HT subgroup compared to control, based on HLA- DRB1/DQB1 haplotypes 9
Table S9. Logistic regressions predicting BGD subgroup compared to control, based on HLA- DRB1/DQB1 haplotypes 10

Comparison between autoimmune thyroid diseases (AITD), Hashimoto's thyroiditis (HT), Basedow-Graves disease (BGD) and control groups, based on genotypes resulting from HLA-DRB1 typing

When analyzing the relationship between HLA-DRB1 genotypes and AITD, HT and BGD, a strong association of the thyroid pathology with the *03/*16 genotype was observed (Tables S1, S2 and S3).

Table S1. Logistic regressions predicting AITD group compared to control, based on HLA-DRB1 genotypes.

HLA-DRB1 genotype	AITD group (n = 77)		Control group (n = 135)		OR (95% C.I.)	*RR (95% C.I)*	*p**
	No.	%	No.	%			
***01/*01**	1	1.30	0	0.00	5.3(0.21-132.2)	2.77(2.31-3.32)	0.36
***01/*03**	2	2.60	0	0.00	8.9(0.42-3.35)	2.8(2.33-3.35)	0.13
***01/*04**	3	3.90	3	2.22	1.78(0.35-9.06)	1.39(0.61-3.16)	0.67
***01/*07**	2	2.60	8	5.93	0.2 (0.02 -1.7)	0.29(0.05-1.9)	0.16
***01/*11**	4	5.19	7	5.19	1(0.28 - 3.54)	1(-0.44 -2.23)	1.00
***01/*13**	2	2.60	6	4.44	0.57(0.11-2. 91)	0.68(0. 20-2.29)	0.71
***01/*15**	3	3.90	2	1.48	2.69(0.44-16.51)	1.67(0.80-3.51)	0.35
***01/*16**	2	2.60	2	1.48	1.77(0.24- 12.85)	1.38(0.51-3.75)	0.62
***03/*03**	0	0.00	1	0.74	0.57(0.02-14.39)	0	1.00
***03/*04**	2	2.60	0	0.00	8.9(0.42-3.35)	2.8(2.33-3.35)	0.13
***03/*07**	0	0.00	2	1.48	0.34(0.01-7.27)	0	0.53
***03/*08**	1	1.30	0	0.00	5.3(0.21-132.2)	2.77(2.31-3.32)	0.36
***03/*09**	0	0.00	1	0.74	0.57(0.02-14.39)	0	1.00
***03/*11**	4	5.19	8	5.93	0.87(0.25 – 2.99)	0.91(-0.40 -2.07)	1.00
***03/*13**	1	1.30	2	1.48	0.87(0.07-9.81)	0.91(0.18-4.5^)	1.00
***03/*14**	0	0.00	2	1.48	0.34(0.01-7.27)	0	0.53
***03/*15**	3	3.90	2	1.48	2.69(0.44-16.51)	1.67(0.80-3.51)	0.35
***03/*16**	**8**	**10.39**	**0**	**0.00**	**33.14(1.88-583.1)**	**2.95(2.44-3.58)**	**0.0002**
***04/*04**	0	0.00	1	0.74	0.57(0.02-14.39)	0	1.00
***04/*07**	1	1.30	0	0.00	5.3(0.21-132.2)	2.77(2.31-3.32)	0.36
***04/*11**	1	1.30	0	0.00	5.3(0.21-132.2)	2.77(2.31-3.32)	0.36
***04/*12**	0	0.00	1	0.74	0.57(0.02-14.39)	0	1.00
***04/*13**	2	2.60	2	1.48	1.77(0.24- 12.85)	1.38(0.51-3.75)	0.62
***04/*15**	2	2.60	5	3.70	0.69(0.13-3.66)	0.78(0.23-2.55)	1.00
***04/*16**	0	0.00	2	1.48	0.34(0.01-7.27)	0	0.53
***07/*07**	1	1.30	1	0.74	1.76(0.1-28.61)	1.38(0.34-5.6)	1.00
***07/*08**	1	1.30	0	0.00	5.3(0.21-132.2)	2.77(2.31-3.32)	0.36
***07/*11**	4	5.19	7	5.19	1(0.28 - 3.54)	1(0.44 -2.23)	1.00
***07/*12**	0	0.00	1	0.74	0.57(0.02-14.39)	0	1.00
***07/*13**	0	0.00	5	3.70	0.15(0.008-2.8)	0	0.16
***07/*14**	3	3.90	1	0.74	5.4(0.55-53.19)	2.1(1.16-3.82)	0.13
***07/*15**	2	2.60	1	0.74	3.57(0.31-40.09)	1.85(0.81-4.22)	0.29
***07/*16**	0	0.00	3	2.22	0.24(0.01-4.79)	0	0.55
***08/*13**	0	0.00	1	0.74	0.57(0.02-14.39)	0	1.00
***08/*14**	0	0.00	1	0.74	0.57(0.02-14.39)	0	1.00
***08/*16**	0	0.00	1	0.74	0.57(0.02-14.39)	0	1.00

***09/*09**	1	1.30	0	0.00	5.3(0.21-132.2)	2.77(2.31-3.32)	0.36
***09/*11**	0	0.00	1	0.74	0.57(0.02-14.39)	0	1.00
***10/*13**	0	0.00	4	2.96	0.18(0.01-3.55)	0	0.29
***10/*15**	1	1.30	0	0.00	5.3(0.21-132.2)	2.77(2.31-3.32)	0.36
***11/*11**	5	6.49	6	4.44	1.49(0.44-5.06)	1.27(0.65 - 2.48)	0.53
***11/*13**	0	0.00	8	5.93	0.09(0.005 - 1.7)	0	0.05
***11/*14**	2	2.60	2	1.48	1.77(0.24- 12.85)	1.38(0.51-3.75)	0.62
***11/*15**	1	1.30	3	2.22	0.57 (0.06-5.66)	0.68(0.12 - 3.77)	1.00
***11/*16**	4	5.19	2	1.48	3.64(0.65 - 20.38)	1.88(1.03 - 3.41)	0.19
***13/*13**	0	0.00	2	1.48	0.34(0.01-7.27)	0	0.53
***13/*14**	0	0.00	2	1.48	0.34(0.01-7.27)	0	0.53
***13/*15**	1	1.30	3	2.22	0.57 (0.06-5.66)	0.68(0.12 - 3.77)	1.00
***13/*16**	2	2.60	4	2.96	0.87(0.15 - 4.88)	0.91(0.29 - 2.88)	1.00
***14/*16**	0	0.00	1	0.74	0.57(0.02-14.39)	0	1.00
***15/*15**	0	0.00	1	0.74	0.57(0.02-14.39)	0	1.00
***15/*16**	2	2.60	3	2.22	1.17(0.19-7.18)	1.10(0.37 - 3.28)	1
***16/*16**	1	1.30	4	2.96	0.43(0.0 5 - 3.92)	0.54(0.09 - 3.17)	0.65

HLA, human leukocyte antigen; AITD, autoimmune thyroid disease; n, persons number; No., genotypes number; OR, odds ratio; CI, confidence interval; RR, risk ratio.

Table S2. Logistic regressions predicting HT subgroup compared to control, based on HLA-DRB1 genotypes.

**HLA-DRB1 genotype**	**HT subgroup (n = 52)**		**Control group (n = 135)**		**OR (95% C.I.)**	***RR(95% C.I)***	***p****
	**No.**	**%**	**No.**	**%**			
***01/*01**	1	1.92	0	0.00	7.89(0.32-197)	3.65(2.89-4.61)	0.28
***01/*03**	2	3.85	0	0.00	13.42(0.63-284.5)	3.70(2.92-4.69)	0.08
***01/*04**	2	3.85	3	2.22	1.76(0.29-10.85)	1.46(0.48-4.37)	0.62
***01/*07**	1	1.92	8	5.93	0.31(0.04-2.55)	0.39(0.06-2.50)	0.45
***01/*11**	2	3.85	7	5.19	0.73(0.15-3.64)	0.79(0.23-2.75)	1.00
***01/*13**	2	3.85	6	4.44	0.86(0.17-4.41)	0.90(0.26-3.04)	1.00
***01/*15**	2	3.85	2	1.48	2.66(0.36-19.41)	1.83(0.67-5.02)	0.31
***01/*16**	1	1.92	2	1.48	1.30(0.11-14.7)	1.2(0.24-6.86)	1.00
***03/*03**	0	0.00	1	0.74	0.85(0.03-21.31)	0	1.00
***03/*04**	1	1.92	0	0.00	7.89(0.32-197)	3.65(2.89-4.61)	0.28
***03/*07**	0	0.00	2	1.48	0.51(0.02-10.78)	0	1.00
***03/*08**	1	1.92	0	0.00	7.89(0.32-197)	3.65(2.89-4.61)	0.28
***03/*09**	0	0.00	1	0.74	0.85(0.03-21.31)	0	1.00
***03/*11**	3	5.77	8	5.93	0.97(0.25-3.82)	0.98(0.36-2.65)	1.00
***03/*13**	0	0.00	2	1.48	0.51(0.02-10.78)	0	1.00
***03/*14**	0	0.00	2	1.48	0.51(0.02-10.78)	0	1.00
***03/*15**	1	1.92	2	1.48	1.30(0.11-14.7)	1.2(0.24-6.86)	1.00
***03/*16**	**3**	**5.77**	**0**	**0.00**	**19.16(0.97-377.9)**	**3.75(2.95-4.78)**	**0.02**
***04/*04**	0	0.00	1	0.74	0.85(0.03-21.31)	0	1.00
***04/*07**	1	1.92	0	0.00	7.89(0.32-197)	3.65(2.89-4.61)	0.28
***04/*11**	1	1.92	10	7.41	0.25(0.03-1.97)	0.31(0.05-2.06)	0.30
***04/*12**	0	0.00	1	0.74	0.85(0.03-21.31)	0	1.00
***04/*13**	2	3.85	2	1.48	2.66(0.36-19.41)	1.83(0.67-5.02)	0.31
***04/*15**	2	3.85	5	3.70	1.04(0.20-5.54)	1.03(0.31-3.4)	1
***04/*16**	0	0.00	2	1.48	0.51(0.02-10.78)	0	1.00
***07/*07**	0	0.00	1	0.74	0.85(0.03-21.31)	0	1.00

***07/*08**	1	1.92	0	0.00	7.89(0.32-197)	3.65(2.89-4.61)	0.28
***07/*11**	2	3.85	7	5.19	0.73(0.15-3.64)	0.79(0.23-2.75)	1.00
***07/*12**	0	0.00	1	0.74	0.85(0.03-21.31)	0	1.00
***07/*13**	0	0.00	5	3.70	0.23(0.01-4.16)	0	0.32
***07/*14**	1	1.92	1	0.74	2.63(0.16-42.83)	1.81(0.44-7.4)	0.48
***07/*15**	2	3.85	1	0.74	5.36(0.48-60.45)	2.45(1.06-5.65)	0.19
***07/*16**	0	0.00	3	2.22	0.36(0.02-7.11)	0	0.56
***08/*13**	0	0.00	1	0.74	0.85(0.03-21.31)	0	1.00
***08/*14**	0	0.00	1	0.74	0.85(0.03-21.31)	0	1.00
***08/*16**	0	0.00	1	0.74	0.85(0.03-21.31)	0	1.00
***09/*09**	1	1.92	0	0.00	7.89(0.32-197)	3.65(2.89-4.61)	0.28
***09/*11**	0	0.00	1	0.74	0.85(0.03-21.31)	0	1.00
***10/*13**	0	0.00	4	2.96	0.28(0.01-5.26)	O	0.58
***10/*15**	1	1.92	0	0.00	7.89(0.32-197)	3.65(2.89-4.61)	0.28
***11/*11**	5	9.62	6	4.44	2.29(0.67-7.85)	1.7(0.85-3.4)	0.18
***11/*13**	0	0.00	8	5.93	0.14(0.01-2.52)	0	0.11
***11/*14**	2	3.85	2	1.48	2.66(0.36-19.41)	1.83(0.67-5.02)	0.31
***11/*15**	1	1.92	3	2.22	8.86(0.09-0.49)	0.9(0.16-4.98)	1
***11/*16**	3	5.77	2	1.48	4.07(0.66-25.11)	2.23(1.05-4.74)	0.13
***13/*13**	0	0.00	2	1.48	0.51(0.02-10.78)	0	1.00
***13/*14**	0	0.00	2	1.48	0.51(0.02-10.78)	0	1.00
***13/*15**	1	1.92	3	2.22	8.86(0.09-0.49)	0.9(0.16-4.98)	1
***13/*16**	1	1.92	4	2.96	0.86(8.87-5.89)	0.71(0.12-4.19)	1
***14/*16**	1	1.92	1	0.74	0.85(0.03-21.31)	0	1.00
***15/*15**	0	0.00	1	0.74	0.85(0.03-21.31)	0	1.00
***15/*16**	2	3.85	3	2.22	1.77(0.29-10.85)	1.46(0.48-4.37)	0.62
***16/*16**	1	1.92	4	2.96	0.86(8.87-5.89)	0.71(0.12-4.19)	1

HLA, human leukocyte antigen; HT, Hashimoto's thyroiditis; n, persons number; No., genotypes number; OR, odds ratio; CI, confidence interval; RR, risk ratio.

Table S3. Logistic regressions predicting BGD subgroup compared to control, based on HLA-DRB1 genotypes.

**HLA-DRB1 genotype**	**BGD subgroup (n = 25)**		**Control group (n = 135)**		**OR (95% C.I.)**	***RR (95% C.I)***	***p****
	**No.**	**%**	**No.**	**%**			
***01/*04**	1	4.00	3	2.22	1.83(0.18-18.38)	1.63(0.27-9.23)	0.5
***01/*07**	0	0.00	8	5.93	0.29(0.02-5.26)	0	0.36
***01/*11**	2	8.00	7	5.19	1.59(0.31-8.14)	1.46(0.41-5.24)	0.63
***01/*13**	0	0.00	6	4.44	0.39(0.02-7.16)	0	0.59
***01/*15**	1	4.00	2	1.48	2.77(0.24-31.79)	2.18(0.42-11.27)	0.4
***01/*16**	1	4.00	2	1.48	2.77(0.24-31.79	2.18(0.42-11.27)	0.4
***03/*03**	0	0.00	1	0.74	1.76(0.07-44.41)	0	1
***03/*04**	1	4.00	0	0.00	16.59(0.66-419.52)	6.63(4.58-9.58)	0.16
***03/*07**	0	0.00	2	1.48	1.05(0.05-22.480	0	1
***03/*09**	0	0.00	1	0.74	1.76(0.07-44.41)	0	1
***03/*11**	1	4.00	8	5.93	0.66(0.08-5.54)	0.70(0.11-4.60)	1
***03/*13**	1	4.00	2	1.48	2.77(0.24-31.79	2.18(0.42-11.27)	0.4
***03/*14**	0	0.00	2	1.48	1.05(0.05-22.480	0	1
***03/*15**	2	8.00	2	1.48	5.78(0.77-43.15)	3.39(1.19-9.69)	0.12
***03/*16**	**5**	**20.00**	**0**	**0.00**	**72.71(3.87-1365.46)**	**7.75(5.15-1.67)**	**<0.0001**
***04/*04**	0	0.00	1	0.74	1.76(0.07-44.41)	0	1

***04/*11**	3	12.00	10	7.41	1.70(0.43-6.69)	1.54(0.57-0.47)	0.43
***04/*12**	0	0.00	1	0.74	1.76(0.07-44.41)	0	1
***04/*13**	0	0.00	2	1.48	1.05(0.05-22.48)	0	1
***04/*15**	0	0.00	5	3.70	0.46(0.02-0.68)	0	1
***04/*16**	0	0.00	2	1.48	1.05(0.05-22.48)	0	1
***07/*07**	1	4.00	1	0.74	5.58(0.34-92.40)	3.29(0.78-13.82)	0.29
***07/*11**	2	8.00	7	5.19	1.59(0.31-8.14)	1.46(0.41-5.24)	0.63
***07/*12**	0	0.00	1	0.74	1.76(0.07-44.41)	0	1
***07/*13**	0	0.00	5	3.70	0.46(0.02-0.68)	0	1
***07/*14**	2	8.00	1	0.74	11.65(1.01-133.88)	4.55(1.88-11.03)	0.06
***07/*15**	0	0.00	1	0.74	1.76(0.07-44.41)	0	1
***07/*16**	0	0.00	3	2.22	0.74(0.04-14.82)	0	1
***08/*13**	0	0.00	1	0.74	1.76(0.07-44.41)	0	1
***08/*14**	0	0.00	1	0.74	1.76(0.07-44.41)	0	1
***08/*16**	0	0.00	1	0.74	1.76(0.07-44.41)	0	1
***09/*11**	0	0.00	1	0.74	1.76(0.07-44.41)	0	1
***10/*13**	0	0.00	4	2.96	0.57(0.03-10.98)	0	1
***11/*11**	0	0.00	6	4.44	0.39(0.02-7.16)	0	0.59
***11/*13**	0	0.00	8	5.93	0.29(0.02-5.27)	0	0.36
***11/*14**	0	0.00	2	1.48	1.05(0.05-22.480	0	1
***11/*15**	0	0.00	3	2.22	0.74(0.04-14.82)	0	1
***11/*16**	1	4.00	2	1.48	2.77(0.24-31.79	2.18(0.42-11.27)	0.4
***13/*13**	0	0.00	2	1.48	1.05(0.05-22.480	0	1
***13/*14**	0	0.00	2	1.48	1.05(0.05-22.480	0	1
***13/*15**	0	0.00	3	2.22	0.74(0.04-14.82)	0	1
***13/*16**	1	4.00	4	2.96	1.36(0.15-12.75)	1.29(0.22-7.75)	0.58
***14/*16**	0	0.00	1	0.74	1.76(0.07-44.41)	0	1
***15/*15**	0	0.00	1	0.74	1.76(0.07-44.41)	0	1
***15/*16**	0	0.00	3	2.22	0.74(0.04-14.82)	0	1
***16/*16**	0	0.00	4	2.96	0.57(0.03-10.98)	0	1

HLA, human leukocyte antigen; BGD, Basedow-Graves' disease; n, persons number; No., genotypes number; OR, odds ratio; CI, confidence interval; RR, risk ratio.

Comparison between autoimmune thyroid diseases (AITD), Hashimoto's thyroiditis (HT), Basedow-Graves disease (BGD) and control groups, based on genotypes resulting from HLA-DQB1 typing

The analysis of the relationship of the HLA-DQB1 genotypes with AITD, HT and BGD did not reveal any significant association with thyroid pathology (Tables S4, S5 and S6).

Table S4. Logistic regressions predicting AITD group compared to control, based on HLA-DQB1 genotypes.

HLA-DQB1 genotype	AITD group (n = 77)		Control group (n = 135)		OR (95% C.I.)	*RR(95% C.I)*	*p**
	No.	%	No.	%			
*02/*02	0	0.00	3	2.22	0.24(0.01-4.79)	0	0.55
*02/*03	12	15.58	15	11.11	1.47(0.65-3.34)	1.27(0.79-2.01)	0.39
*02/*04	1	1.30	0	0.00	5.31(0.21-132.2)	2.77(2.32-3.32)	0.36
*02/*05	12	15.58	12	8.89	1.89(0.8-4.45)	1.45(0.93-2.26)	0.18
*02/*06	6	7.79	7	5.19	1.55(0.50-4.78)	1.29(0.70-2.40)	0.55
*03/*03	11	14.29	29	21.48	0.61(0.29-1.30)	0.72(0.42-2.23)	0.27
*03/*04	0	0.00	0	0.00	0	0	0
*03/*05	15	19.48	20	14.81	1.39(0.67-2.91)	1.22(0.79-1.89)	0.44
*03/*06	6	7.79	16	11.85	0.63(0.24-1.68)	0.73(0.36-1.48)	0.48
*04/*04	0	0.00	0	0.00	0	0	0
*04/*05	0	0.00	2	1.48	0.34(0.02-728)	0	0.54
*04/*06	0	0.00	0	0.00	0	0	0
*05/*05	5	6.49	7	5.19	1.27(0.39-4.15)	1.16(0.58-2.32)	0.76
*05/*06	9	11.69	20	14.81	0.76(0.33-1.77)	0.84(0.47-1.48)	0.68
*06/*06	0	0.00	4	2.96	0.19(0.01-3.55)	0	0.30

HLA, human leukocyte antigen; AITD, autoimmune thyroid disease; n, persons number; No., genotypes number; OR, odds ratio; CI, confidence interval; RR, risk ratio.

Table S5. Logistic regressions predicting HT subgroup compared to control, based on HLA-DQB1 genotypes.

HLA-DQB1 genotype	HT subgroup (n = 52)		Control group (n = 135)		OR (95% C.I.)	*RR(95% C.I)*	*p**
	No.	%	No.	%			
*02/*02	0	0	3	2.22	0.36(0.02-7.11)	0	0.56
*02/*03	7	13.46	15	11.11	1.24(0.48-3.25)	1.17(0.60-2.26)	0.62
*02/*04	1	1.92	0	0.00	7.89(0.32-197.0)	3.65(2.89-4.61)	0.28
*02/*05	7	13.46	12	8.89	1.59(0.59-4.30)	1.38(0.73-2.61)	0.42
*02/*06	3	5.77	7	5.19	1.12(0.28-4.51)	1.08(0.41-2.88)	1.00
*03/*03	8	15.38	29	21.48	0.66(0.28-1.57)	0.74(0.38-1.43)	0.42
*03/*04	0	0.00	0	0.00	0	0	0
*03/*05	9	17.31	20	14.81	1.20(0.51-2.85)	1.14(0.63-2.08)	0.66
*03/*06	6	11.54	16	11.85	0.97(0.36-2.63)	0.98(0.47-2.82)	1.00
*04/*04	0	0.00	0	0.00	0	0	0
*04/*05	0	0.00	2	1.48	0.51(0.02-10.78)	0	1.00
*04/*06	0	0.00	0	0.00	0	0	0
*05/*05	4	7.69	7	5.19	1.52(0.43-5.44)	1.33(0.59-3.02)	0.50
*05/*06	7	13.46	20	14.81	0.89(0.35-2.26)	0.92(0.47-1.83)	1.00
*06/*06	0	0.00	4	2.96	0.27(0.01-5.26)	0	0.58

HLA, human leukocyte antigen; HT, Hashimoto's thyroiditis; n, persons number; No., genotypes number; OR, odds ratio; CI, confidence interval; RR, risk ratio.

Table S6. Logistic regressions predicting BGD subgroup compared to control, based on HLA-DQB1 genotypes.

HLA-DQB1 genotype	BGD subgroup (n = 25)		Control group (n = 135)		OR (95% C.I.)	*RR(95% C.I)*	*p**
	No.	%	No.	%			
***02/*02**	0	0	3	2.22	0.74(0.04-14.82)	0	1.00
***02/*03**	5	20.00	15	11.11	2.00(0.65-6.12)	1.75(0.74-4.14)	0.32
***02/*04**	0	0.00	0	0.00	0	0	0
***02/*05**	5	20.00	12	8.89	2.56(0.52-8.06)	2.10(0.91-4.88)	0.15
***02/*06**	3	12.00	7	5.19	2.49(0.60-1.38)	2.05(0.74-5.69)	0.19
***03/*03**	3	12.00	29	21.48	0.50(0.14-1.78)	0.55(0.17-1.71)	0.41
***03/*04**	0	0.00	0	0.00	0	0	0
***03/*05**	6	24.00	20	14.81	1.82(0.65-5.11)	1.63(0.72-3.68)	0.25
***03/*06**	0	0.00	16	11.85	0.14(0.01-2.45)	0	0.08
***04/*04**	0	0.00	0	0.00	0	0	0
***04/*05**	0	0.00	2	1.48	1.05(0.05-22.48)	0	1.00
***04/*06**	0	0.00	0	0.00	0	0	0
***05/*05**	1	4.00	7	5.19	0.76(0.09-6.48)	0.79(0.12-5.14)	1.00
***05/*06**	2	8.00	20	14.81	0.5(0.11-2.29)	0.55(0.14-2.15)	0.53
***06/*06**	0	0.00	4	2.96	0.57(0.03-10.98)	0	1.00

HLA, human leukocyte antigen; BGD, Basedow-Graves' disease; n, persons number; No., genotypes number; OR, odds ratio; CI, confidence interval; RR, risk ratio.

Comparison between autoimmune thyroid diseases (AITD), Hashimoto's thyroiditis (HT), Basedow-Graves disease (BGD) and control groups, based on HLA-DRB1/DQB1 haplotypes resulting

The association of HLA-DRB1/DQB1 haplotypes with AITD, HT and GD (Tables S7, S8, S9) revealed a significant positive association for the DRB1*03/DQB1*06 haplotype in the BGD subgroup.

Table S7. Logistic regressions predicting AITD group compared to control, based on HLA-DRB1/DQB1 haplotypes.

HLA-DRB1/DQB1 haplotype	AITD group (n = 77)		Control group (n = 135)		OR (95% C.I.)	RR (95% C.I)	*p**
	No.	%	No.	%			
**DRB1*01/DQB1*02**	3	3.90	7	5.19	0.74(0.19-2.95)	0.82(0.31-2.15)	0.75
**DRB1*01/DQB1*03**	7	9.09	12	8.89	1.03(0.39-2.72)	1.02(0.55-1.88)	1.00
**DRB1*01/DQB1*04**	-	-	-	-	-	-	-
**DRB1*01/DQB1*05**	8	10.39	27	20.00	1.22(0.62-2.40)	1.13(0.75-1.71)	0.60
**DRB1*01/DQB1*06**	4	5.19	7	5.19	1.00(0.28-3.54)	1.00(0.45-2.24)	1.00
**DRB1*03/DQB1*02**	11	14.29	16	11.85	1.24(0.54 -2.83)	1.14(0.70-1.87)	0.67
**DRB1*03/DQB1*03**	6	7.79	10	7.41	1.06(0.37-3.03)	1.04(0.54-2.00)	1.00
**DRB1*03/DQB1*05**	0	0.00	4	2.96	0.19(0.01-3.55)	0	0.30
**DRB1*03/DQB1*06**	4	5.19	3	2.22	2.41(0.53-11.07)	1.60(0.87-3.13)	0.26
**DRB1*04/DQB1*02**	2	2.60	0	0.00	8.97(0.42-189.50)	2.80(2.34-3.36)	0.13
**DRB1*04/DQB1*03**	14	18.18	24	17.78	1.03(0.50-2.13)	1.02(0.64-1.61)	1.00
**DRB1*04/DQB1*05**	2	2.60	5	3.70	0.69(0.13-3.66)	0.78(0.24-2.56)	1.00
**DRB1*04/DQB1*06**	4	5.19	7	5.19	1.00(0.28-3.56)	1.00(0.45-2.24)	1.00
**DRB1*07/DQB1*02**	10	12.99	20	14.81	0.86(0.38-1.94)	0.91(0.53-1.56)	0.84
**DRB1*07/DQB1*03**	8	10.39	13	9.63	1.09(0.43-2.76)	1.05(0.59-1.88)	1.00
**DRB1*07/DQB1*04**	1	1.30	0	0.00	5.31(0.21-132.15)	2.78(2.32-3.32)	0.36
**DRB1*07/DQB1*05**	4	5.19	7	5.19	1.00(0.28-3.56)	1.00(0.45-2.24)	1.00
**DRB1*07/DQB1*06**	2	2.60	6	4.44	0.57(0.11-2.91)	0.68(0.02-2.29)	0.71
**DRB1*08/DQB1*02**	2	2.60	0	0.00	8.97(0.42-189.50)	2.80(2.34-3.36)	0.13
**DRB1*08/DQB1*03**	1	1.30	1	0.74	1.76(0.11-28.61)	1.38(0.34-5.59)	1.00
**DRB1*08/DQB1*04**	1	1.30	2	1.48	0.88(0.08-9.82)	0.92(0.18-4.59)	1.00
**DRB1*08/DQB1*05**	0	0.00	2	1.48	0.34(0.02-7.27)	0	0.54
**DRB1*08/DQB1*06**	0	0.00	1	0.74	0.58(0.02-14.39)	0	1.00
**DRB1*09/DQB1*02**	0	0.00	1	0.74	0.58(0.02-14.39)	0	1.00
**DRB1*09/DQB1*03**	1	1.30	2	1.48	0.88(0.08-9.82)	0.92(0.18-4.59)	1.00
**DRB1*10/DQB1*05**	1	1.30	3	2.22	0.58(0.06-5.67)	0.68(0.12-3.78)	1.00
**DRB1*10/DQB1*06**	1	1.30	3	2.22	0.58(0.06-5.67)	0.68(0.12-3.78)	1.00
**DRB1*11/DQB1*02**	8	10.39	10	7.41	1.45(0.55-3.84)	1.25(0.72-2.17)	0.45
**DRB1*11/DQB1*03**	28	36.36	54	40.00	0.86(0.48-1.53)	0.91(0.62-1.32)	0.66
**DRB1*11/DQB1*05**	10	12.99	12	8.89	1.53(0.63-7.73)	1.29(0.78-2.12)	0.36
**DRB1*11/DQB1*06**	1	1.30	6	4.44	0.28(0.03-2.40)	0.39(0.06-2.39)	0.43
**DRB1*13/DQB1*03**	0	0.00	8	5.93	0.10(0.01-1.70)	0	0.053
**DRB1*13/DQB1*06**	0	0.00	3	2.22	0.24(0.01-4.79)	0	0.56

**DRB1*14/DQB1*03**	2	2.60	2	1.48	1.77(0.24-12.85)	1.39(0.51-3.76)	0.62
**DRB1*14/DQB1*05**	2	2.60	2	1.48	1.77(0.24-12.85)	1.39(0.51-3.76)	0.62
**DRB1*15/DQB1*03**	1	1.30	3	2.22	0.58(0.06-5.67)	0.68(0.12-3.78)	1.00
**DRB1*15/DQB1*06**	1	1.30	3	2.22	0.58(0.06-5.67)	0.68(0.12-3.78)	1.00
**DRB1*16/DQB1*03**	4	5.19	2	1.48	3.64(0.65-20.38)	1.88(1.04-3.41)	0.19
**DRB1*16/DQB1*05**	4	5.19	2	1.48	3.64(0.65-20.38)	1.88(1.04-3.41)	0.19

HLA, human leukocyte antigen; AITD, autoimmune thyroid disease; n, persons number; No., haplotypes number; OR, odds ratio; CI, confidence interval; RR, risk ratio.

Table S8.Logistic regressions predicting HT subgroup compared to control, based on HLA-DRB1/DQB1 haplotypes.

**HLA-DRB1/DQB1 haplotype**	**HT subgroup (n = 52)**		**Control group (n = 135)**		**OR (95% C.I.)**	**RR (95% C.I)**	***p****
	**No.**	**%**	**No.**	**%**			
**DRB1*01/DQB1*02**	3	5.77	7	5.19	1.11(0.27-4.5)	1.08(0.4-2.87)	1
**DRB1*01/DQB1*03**	4	7.69	12	8.89	0.85(0.26-2.77)	0.89(0.36-2.15)	1
**DRB1*01/DQB1*04**	-	-	-	-	-	-	-
**DRB1*01/DQB1*05**	13	25.00	27	20.00	1.33(0.62-2.84)	1.22(0.72-2.06)	0.55
**DRB1*01/DQB1*06**	6	11.54	7	5.19	1.11(0.27-4.5)	1.08(0.4-2.87)	1
**DRB1*03/DQB1*02**	6	11.54	16	11.85	0.97(0.36-2.63)	0.98(0.47-2.02)	1
**DRB1*03/DQB1*03**	4	7.69	10	7.41	1.04(0.31-3.48)	1.03(0.43-2.43)	1
**DRB1*03/DQB1*05**	0	0.00	4	2.96	0.28(0.01-5.26)	0	0.58
**DRB1*03/DQB1*06**	1	1.92	3	2.22	0.86(0.09-8.49)	0.90(0.16-4.98)	1.00
**DRB1*04/DQB1*02**	1	1.92	0	0.00	7.89(0.32-197.05)	3.65(2.89-4.61)	0.28
**DRB1*04/DQB1*03**	9	17.31	24	17.78	0.97(0.42-2.25)	0.98(0.53-1.8)	1
**DRB1*04/DQB1*05**	1	1.92	5	3.70	0.51(0.06-4.47)	0.59(0.10-3.60)	1
**DRB1*04/DQB1*06**	4	7.69	7	5.19	1.52(0.43-5.44)	1.33(0.59-3.02)	0.50
**DRB1*07/DQB1*02**	7	13.46	20	14.81	0.89(0.35-2.26)	0.92(0.47-1.83)	1
**DRB1*07/DQB1*03**	5	9.62	13	9.63	1(0.33-2.95)	1(0.45-2.18)	1
**DRB1*07/DQB1*04**	1	1.92	0	0.00	7.89(0.32-197.05)	3.65(2.89-4.61)	0.28
**DRB1*07/DQB1*05**	2	3.85	7	5.19	0.73(0.14-3.64)	0.79(0.23-2.75)	1
**DRB1*07/DQB1*06**	2	3.85	6	4.44	0.86(0.17-4.41)	0.90(0.26-3.04)	1
**DRB1*08/DQB1*02**	2	3.85	0	0.00	13.42(0.63-284.49)	3.70(2.92-4.69)	0.08
**DRB1*08/DQB1*03**	1	1.92	1	0.74	2.63(0.16-42.83)	1.81(0.44-7.40)	0.48
**DRB1*08/DQB1*04**	1	1.92	2	1.48	1.3(0.12-14.70)	1.20(0.24-6.06)	1
**DRB1*08/DQB1*05**	0	0.00	2	1.48	0.5(0.02-10.78)	0	1
**DRB1*08/DQB1*06**	0	0.00	1	0.74	0.85(0.03-21.31)	0	1
**DRB1*09/DQB1*02**	0	0.00	1	0.74	0.85(0.03-21.31)	0	1
**DRB1*09/DQB1*03**	1	1.92	2	1.48	1.3(0.12-14.70)	1.20(0.24-6.06)	1
**DRB1*10/DQB1*05**	1	1.92	3	2.22	0.86(0.09-8.49)	0.90(0.16-4.98)	1.00
**DRB1*10/DQB1*06**	1	1.92	3	2.22	0.86(0.09-8.49)	0.90(0.16-4.98)	1.00
**DRB1*11/DQB1*02**	5	9.62	10	7.41	1.33(0.43-4.10)	1.22(0.57-2.60)	0.76
**DRB1*11/DQB1*03**	19	36.54	54	40.00	0.86(0.45-1.67)	0.9(0.56-1.46)	0.74
**DRB1*11/DQB1*05**	7	13.46	12	8.89	1.59(0.59-4.30)	1.38(0.73-2.61)	0.42
**DRB1*11/DQB1*06**	1	1.92	6	4.44	0.42(0.05-3.59)	0.5(0.08-3.14)	0.68
**DRB1*13/DQB1*03**	0	0.00	8	5.93	0.14(0.01-2.52)	0	0.11
**DRB1*13/DQB1*06**	0	0.00	3	2.22	0.36(0.02-7.11)	0	0.56
**DRB1*14/DQB1*03**	2	3.85	2	1.48	2.66(0.36-19.41)	1.83(0.67-5.02)	0.31
**DRB1*14/DQB1*05**	2	3.85	2	1.48	2.66(0.36-19.41)	1.83(0.67-5.02)	0.31
**DRB1*15/DQB1*03**	1	1.92	3	2.22	0.86(0.09-8.49)	0.90(0.16-4.98)	1.00
**DRB1*15/DQB1*06**	1	1.92	3	2.22	0.86(0.09-8.49)	0.90(0.16-4.98)	1.00
**DRB1*16/DQB1*03**	3	5.77	2	1.48	4.07(0.66-25.11)	2.23(1.50-4.74)	0.13
**DRB1*16/DQB1*05**	3	5.77	2	1.48	4.07(0.66-25.11)	2.23(1.50-4.74)	0.13

HLA, human leukocyte antigen; HT, Hashimoto's thyroiditis; n, persons number; No., haplotypes number; OR, odds ratio; CI, confidence interval; RR, risk ratio.

Table S9. Logistic regressions predicting BGD subgroup compared to control, based on HLA-DRB1/DQB1 haplotypes.

HLA-DRB1/DQB1 haplotype	BGD subgroup (n = 25)		Control group (n = 135)		OR (95% C.I.)	RR (95% C.I)	*p**
	No.	%	No.	%			
**DRB1*01/DQB1*02**	0	0.00	7	5.19	0.33(0.02-6.07)	0	0.6
**DRB1*01/DQB1*03**	3	12.00	12	8.89	1.40(0.36-5.36)	1.32(0.45-3.89)	0.71
**DRB1*01/DQB1*04**	-	-	-	-	-	-	-
**DRB1*01/DQB1*05**	5	20.00	27	20.00	1(0.34-2.91)	1(0.41-2.46)	1.00
**DRB1*01/DQB1*06**	1	4.00	7	5.19	0.76(0.09-6.48)	0.79(0.12-5.14)	1
**DRB1*03/DQB1*02**	5	20.00	16	11.85	0.86(0.61-5.63)	1.65(0.70-3.93)	0.33
**DRB1*03/DQB1*03**	2	8.00	10	7.41	1.09(0.22-5.29)	1.07(0.29-4.01)	1.00
**DRB1*03/DQB1*05**	0	0.00	4	2.96	0.57(0.03-10.98)	0	1
**DRB1*03/DQB1*06**	**3**	**12.00**	**3**	**2.22**	**6.00(1.14-31.66)**	**3.5(1.44-8.51)**	**0.049**
**DRB1*04/DQB1*02**	1	4.00	0	0.00	16.59(0.66-419.52)	0.63(4.58-9.58)	0.16
**DRB1*04/DQB1*03**	5	20.00	24	17.78	1.16(0.39-3.39)	1.13(0.46-2.76)	0.78
**DRB1*04/DQB1*05**	1	4.00	5	3.70	1.08(0.12-9.69)	1.07(0.17-6.65)	1
**DRB1*04/DQB1*06**	0	0.00	7	5.19	0.34(0.02-6.07)	0	0.60
**DRB1*07/DQB1*02**	2	8.00	20	14.81	0.78(0.21-2.87)	0.81(0.26-2.50)	1.00
**DRB1*07/DQB1*03**	3	12.00	13	9.63	1.28(0.34-4.86)	1.23(0.41-3.65)	0.72
**DRB1*07/DQB1*04**	-	-	-	-	-	-	-
**DRB1*07/DQB1*05**	2	8.00	7	5.19	1.59(0.31-8.14)	1.46(0.41-5.24)	0.63
**DRB1*07/DQB1*06**	0	0.00	6	4.44	0.39(0.02-7.16)	0	0.59
**DRB1*08/DQB1*02**	-	-	-	-	-	-	-
**DRB1*08/DQB1*03**	0	0.00	1	0.74	1.76(.07-44.41)	0	1.00
**DRB1*08/DQB1*04**	0	0.00	2	1.48	1.05(0.05-22.48)	0	1.00
**DRB1*08/DQB1*05**	0	0.00	2	1.48	1.05(0.05-22.48)	0	1.00
**DRB1*08/DQB1*06**	0	0.00	1	0.74	1.76(.07-44.41)	0	1.00
**DRB1*09/DQB1*02**	0	0.00	1	0.74	1.76(.07-44.41)	0	1.00
**DRB1*09/DQB1*03**	0	0.00	2	1.48	1.05(0.05-22.48)	0	1.00
**DRB1*10/DQB1*05**	0	0.00	3	2.22	0.74(0.04-14.82)	0	1.00
**DRB1*10/DQB1*06**	0	0.00	3	2.22	0.74(0.04-14.82)	0	1.00
**DRB1*11/DQB1*02**	3	12.00	10	7.41	1.70(0.43-6.69)	1.54(0.53-4.74)	0.43
**DRB1*11/DQB1*03**	9	36.00	54	40.00	0.84(0.53-2.05)	0.87(0.41-1.84)	0.82
**DRB1*11/DQB1*05**	3	12.00	12	8.89	1.40(0.36-5.36)	1.32(0.45-3.89)	0.71
**DRB1*11/DQB1*06**	0	0.00	6	4.44	0.39(0.02-7.16)	0	0.59
**DRB1*13/DQB1*03**	0	0.00	8	5.93	0.29(0.02-5.26)	0	0.36
**DRB1*13/DQB1*06**	0	0.00	3	2.22	0.74(0.04-14.82)	0	1.00
**DRB1*14/DQB1*03**	0	0.00	2	1.48	1.05(0.05-22.48)	0	1.00
**DRB1*14/DQB1*05**	0	0.00	2	1.48	1.05(0.05-22.48)	0	1.00
**DRB1*15/DQB1*03**	0	0.00	3	2.22	0.74(0.04-14.82)	0	1.00
**DRB1*15/DQB1*06**	0	0.00	3	2.22	0.74(0.04-14.82)	0	1.00
**DRB1*16/DQB1*03**	1	4.00	2	1.48	2.77(0.24-31.79)	2.18(0.42-11.27)	0.40
**DRB1*16/DQB1*05**	1	4.00	2	1.48	2.77(0.24-31.79)	2.18(0.42-11.27)	0.40

HLA, human leukocyte antigen; BGD, Basedow-Graves' disease; n, persons number; No., haplotypes number; OR, odds ratio; CI, confidence interval; RR, risk ratio.
